# The Role of Translation Initiation Regulation in Haematopoiesis

**DOI:** 10.1155/2012/576540

**Published:** 2012-05-09

**Authors:** Godfrey Grech, Marieke von Lindern

**Affiliations:** ^1^Department of Pathology, Medical School, University of Malta, Msida MSD 2090, Malta; ^2^Department of Hematopoiesis, Sanquin Research and Landsteiner Laboratory, AMC/UvA, 1066 CX Amsterdam, The Netherlands

## Abstract

Organisation of RNAs into functional subgroups that are translated in response to extrinsic and intrinsic factors underlines a relatively unexplored gene expression modulation that drives cell fate in the same manner as regulation of the transcriptome by transcription factors. Recent studies on the molecular mechanisms of inflammatory responses and haematological disorders indicate clearly that the regulation of mRNA translation at the level of translation initiation, mRNA stability, and protein isoform synthesis is implicated in the tight regulation of gene expression. This paper outlines how these posttranscriptional control mechanisms, including control at the level of translation initiation factors and the role of RNA binding proteins, affect hematopoiesis. The clinical relevance of these mechanisms in haematological disorders indicates clearly the potential therapeutic implications and the need of molecular tools that allow measurement at the level of translational control. Although the importance of miRNAs in translation control is well recognised and studied extensively, this paper will exclude detailed account of this level of control.

## 1. Introduction

Hematopoietic stem cells (HSCs) have a life-long capacity to replenish the stem cell compartment and give rise to multipotent progenitors. These progenitors expand to maintain the hematopoietic compartment and differentiate into various blood lineage progenitors. Lineage positive progenitors are committed for differentiation into mature blood cells.

Transcription factors have a pivotal role in hematopoiesis to maintain a gene expression program that endows self-renewal properties to HSCs and enables commitment and differentiation into different blood cell lineages [1]. The upregulation of both PU.1 and Gata-1 reprograms HSC to become common myeloid progenitors (CMPs) [2]. The CMPs undergo further lineage divergence into megakaryocyte/erythroid progenitors (MEPs) and granulocyte/monocyte progenitors (GMPs) upon Gata-1 and PU.1 mutual exclusive expression, respectively. Commitment to the erythroid lineage is characterized by the expression of erythroid-specific transcription factors Gata-1, Eklf, and Nfe2 determining the erythroid program.

Upon commitment, the balance between proliferation and differentiation of lineage-specific progenitors is under tight control, to maintain the progenitor pool and ensure maturation in response to physiological demand. The production of increased numbers of mature blood cells during stress situations such as inflammation or hypoxia requires higher progenitor proliferation rates. Concurrently, feedback mechanisms must be closely coordinated to repress progenitor proliferation when the stress is over [3]. The human bone marrow must replace 10^11^ erythrocytes daily under normal physiological erythropoiesis. Gene expression is regulated at the transcription level, producing a cell-specific mRNA pool. Subsequent control of mRNA translation enables the cells to further adapt to environmental and developmental cues. Translation regulation (i) permits fast cellular responses to growth factors, inducing specific proteins to be expressed, (ii) selective expression of different protein isoforms from a given transcript, and (iii) induction of expression of pro-apoptotic proteins when the transcription program is inhibited. Translation Initiation is an important level of translation control. Cap-dependent translation initiation depends on two major limiting steps: (i) the formation of the initiation complex by release of the cap-binding initiation factor eIF4E from its binding factor 4E-BP and (ii) binding of the ternary complex (TC) consisting of GTP-loaded translation initiation factor 2 (eIF2:GTP) plus a methionine-loaded initiator tRNA (tRNA_i_^met^) to the 40S ribosome subunit. Cap-independent translation initiation depends on an internal ribosomal entry site (IRES) in the transcript that has to bind IRES transactivating factors (ITAFs). The biochemistry of translation initiation has been extensively reviewed [4, 5]. We will focus on the importance of translation initiation for haematopoiesis.

## 2. Growth Factor-Dependent Proliferation of Hematopoietic Progenitors

The main regulator of erythropoiesis is the glycoprotein hormone Erythropoietin (Epo), produced in the kidney in response to oxygen tension in the blood. The function of Epo initiates from the specific interaction to its cell surface receptor (EpoR). In stress erythropoiesis, stem cell factor (cKit ligand) and glucocorticoids (GR) work in concert with Epo to induce expansion of progenitors in the mouse spleen [6, 7]. The requirement for SCF in acute erythroid expansion was demonstrated by the observation that inhibiting c-Kit antibodies abolished splenic hematopoiesis upon induction of haemolytic anaemia in mice, while the antibodies had no effect on steady-state erythropoiesis [6].

**Figure 1:**
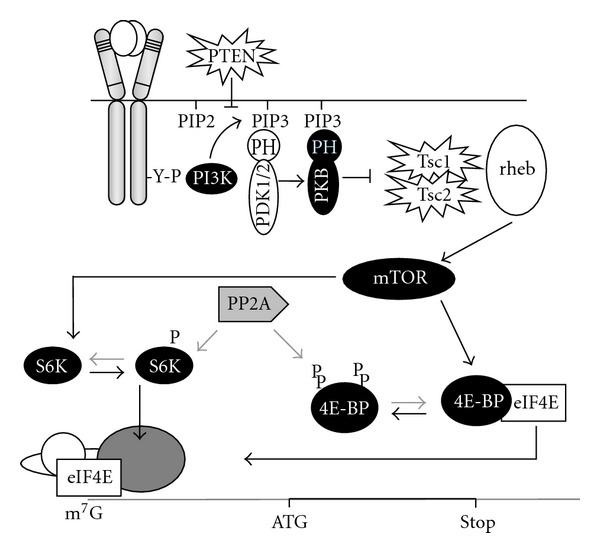
The PI3K/PKB/mTOR pathway controls mRNA translation. SCF-receptor activation results in recruitment of PI3K to the receptor, which generates phosphorylates membrane lipids (PIP3) that form an anchor for the PH-domain containing kinases PDK1 and PKB. PIP3 is dephosphorylated by the tumour suppressor PTEN, which silences the PI3K-pathway. At the membrane PDK1 phosphorylates PKB, which phosphorylates the tuberous sclerosis tumour suppressor genes Tsc1 and Tsc2. Upon phosphorylation these genes release the GTPase Rheb to activate mTOR. Activation of mTOR results in phosphorylation of p70S6kinase (S6K) and eIF4E-binding protein (4E-BP). Upon phosphorylation, 4E-BP releases the cap-binding translation initiation factor 4E (eIF4E), which allows for association of eIF4E with the proteins that form the eIF4F scanning complex and with the 40S ribosomal subunit [4].

Epo and SCF transduce signals via multiple cooperating pathways in erythroid progenitors [7–10], among which the activation of the PI3K pathway. Although both Epo and SCF activate PI3K in erythroid progenitors, the efficiency with which downstream signalling pathways are activated shows large differences [11, 12], suggesting differential susceptibility to feedback pathways. Activation of PI3K results in phosphorylation and activation of PKB and subsequently of mTOR (Figure 1). In turn, mTOR phosphorylates and activates S6K (Rps6kb1; p70S6Kinase) and 4EBP (4E-Binding Protein) [13]. In erythroblasts, only SCF can induce full phosphorylation of 4EBP [14]. PP2A is the main phosphatase acting on S6K and 4EBP1 and thereby the main antagonist of mTOR function in erythroblasts (Figure 1).

4EBP hyperphosphorylation results in the release of the mRNA cap-binding factor eIF4E (eukaryotic Initiation Factor 4E) [15]. Subsequently, eIF4E can bind the scaffold protein eIF4G, which enables the formation of an eIF4F scanning complex containing eIF4E, eIF4G, and the RNA helicase eIF4A. eIF4F associates with several other translation factors including the 40S small subunit of the ribosome and the associated ternary complex consisting of eIF2:tRNA_i_^met^ [5]. This preinitiation complex scans the 5′UTR for the first AUG codon in an appropriate sequence context [16]. There the ribosome associates and methionine is deposited at the P-site and eIF2 is released [5, 17]. The cap-binding eIF4E protein is a rate limiting factor in the formation of the preinitiation complex [18] and therefore its release upon phosphorylation of 4EBP is a crucial control mechanism in polysome recruitment of mRNAs. In addition, eIF4E is phosphorylated by MAP-kinase signal-integrating kinases Mnk1 and Mnk2 [19, 20] in response to insulin and stress [21]. The role of eIF4E phosphorylation is still controversial.

### 2.1. Translation Initiation Sensitivity to eIFs: Regulation of eIF4F-Sensitive Transcripts

Although the preinitiation complex consists of general translation factors, it scans mRNAs with a short and simple 5′ Untranslated Region (UTR) much more efficiently than mRNAs with a long and structured 5′UTR [22]. Structured mRNAs require a higher density of preinitiation complexes to maintain an open structure, which renders them more sensitive to the concentration of eIF4F complexes in the cell. Well-known examples are growth promoting proteins such as VEGF, MYC, and ODC [22]. PI3K-dependent, selective polysome recruitment of mRNA is important in v-Ras/v-Akt-transformed glioblastoma cells [23] and in metastasis of human epithelial cells [24]. Transcripts that are specifically recruited to polysomes upon overexpression of eIF4E have been identified in mouse and human cells [25, 26].

Proliferation of erythroid progenitors under conditions that mimic stress erythropoiesis is strictly dependent on PI3K activity [11, 27]. Overexpression of eIF4E abrogated the requirement for SCF-induced PI3K activation, which suggested a role of selective polysome recruitment of transcript with a complex RNA structure [12]. Genomewide profiling of both total and polysome-associated mRNAs in an erythroblast cell line, I/11, cultured in presence or absence of growth factors identified a large number of constitutively expressed transcripts that are selectively translated in response to PI3K activation, or upon overexpression of eIF4E [14]. This list of eIF4E-sensitive transcripts included alpha4, a subunit of protein phosphatase 2a (PP2a). PP2A exists in various complexes that shift target specificity depending on the binding of regulatory components. mTOR modulates the formation of the PP2A-*α*4 complex sequestrating the phosphatase activity away from its own downstream targets 4EBP and S6K [28–31]. Constitutive expression of alpha4 in erythroid progenitors completely blocked erythroid differentiation and endowed erythroblast with long-term proliferation in the absence of SCF. It maintained the activation state of the mTOR targets 4EBP and p70S6k in the presence of Epo alone and enhanced polysome recruitment of other eIF4E-senstive transcripts [14]. This further underlines the importance of eIF4E-dependent translation and of the proteins controlled by this mechanism. Among them are indeed several proteins that are essential for erythropoiesis such as Nme2 [14, 32, 33] and the SNARE protein Use1 that is essential for retrograde transport of vesicles from the Golgi to the ER [34].

 Mutations that enhance translation initiation efficiency have been implicated in the aggressiveness of various human cancers including Acute Myeloid Leukemia (AML) [35, 36]. Constitutive activation of PI3K and mTOR occurs at high frequency in AML [37]. Mostly the cause of constitutive PI3K activation is unknown, but mutations in the receptor kinases cKIT and FLT3 are candidates. The D816V mutation in the kinase domain of cKit activates the PI3K/PKB/mTOR pathway and confers sensitivity to rapamycin [38]. Interestingly, rapamycin induces cell cycle arrest and apoptosis in patient-derived neoplastic mast cells harbouring the D816V *cKIT*, but not in normal human cord-blood-derived mast cells. This implies that inhibitors targeting translation initiation regulators are therapeutic candidates in the treatment of aggressive systemic mastocytosis (associated with *cKIT* D816V) and in AML harbouring the D816V *cKIT* mutant (present in 10 to 40% of core-binding factor leukaemia [39]).

Enhanced eIF4E-dependent translation of transcripts with a structured 5′UTR also contributes to chronic myelogenous leukaemia (CML). Leukemic transformation of hematopoietic progenitors by the BCR/ABL fusion protein depends on PI3K activation, which will enhance 4EBP phosphorylation and eIF4E release. In addition, BCR/ABL induces expression of SET, which subsequently acts as an inhibitory regulatory subunit of PP2A similar to *α*4 [14, 40]. Notably, the leukaemic potential of BCR/ABL-expressing cells can be inhibited by pharmacological activation of the phosphatase pp2a [41].

Pharmacological inactivation of mTOR with rapamycin reduces neoplastic proliferation in PTEN deficient mice, and reverses tumour growth in cancer cells characterised by activated PKB [42]. In AML, however, trials with rapamycin have been put on halt because rapamycin induced a feedback pathway resulting in further PKB activation [43]. Given the role of the Pp2a regulatory subunit *α*4, reactivation of the phosphatase, pp2a, offers a potential alternative treatment to therapy-resistant patients [44].

### 2.2. Translation Initiation Sensitivity to eIFs: eIF2*α* Phosphorylation and AUG Selection

The second limiting translation factor next to eIF4E is eIF2, a GTPase that is associated with methionine-loaded initiator tRNA only in its GTP-bound state. This complex of eIF2:GTP/tRNA_i_^met^ is known as the ternary complex (TC) [5]. The GTPase activity of eIF2 is activated by recognition of an AUG codon and depends on the sequence context. The better an initiation codon resembles the consensus sequence (A/GnnAUGG), the higher the chance that the eIF2 GTPase activity is triggered and methionine is delivered to the P-site of the ribosome [45]. This then leads to release of eIF2:GDP. In addition to availability, also the activity of eIF2 is regulated. The initiation factor eIF5 increases GTPase activity and increases the probability that methionine is deposited at the P-site of the ribosome at a start codon in a less optimal context [17]. The eIF2 is a trimeric protein that is regulated by phosphorylation of the eIF2*α* subunit. Phosphorylation inhibits the recovery of eIF2:GTP from eIF2:GDP by protein eIF2B, and thereby the reassociation with tRNA_i_^met^. The kinases involved in eIF2 phosphorylation are activated by lack of haem (HRI), unfolded proteins in the ER (PERK), double strand RNA (RNA viruses; PKR), or lack of amino acids (GCN2) [46]. In the hematopoietic system, HRI is extremely important in erythropoiesis because it coordinates haemoglobin synthesis. It links iron availability and haem synthesis to the translation of globin chains [47]. Phosphorylation of eIF2 results in translation of Atf4 (activating transcription factor 4) and subsequent transcription of Gadd34. Gadd34 is an activating regulatory subunit of phosphatase Pp1 that is able to dephosphorylate eIF2-P. Mice lacking this feedback regulation develop severe anemia [48].

Similar to eIF4E, also eIF2 availability has consequences for both the overall protein synthesis rate and the translation of transcripts that carry regulatory sequences in their 5′UTR. The phosphorylation level of eIF2 specifically controls translation of transcripts with upstream open reading frames (uORFs). Following the translation of a short uORF, the preinitiation complex lacking eIF2 continues scanning. During scanning the TC will reassociate to enable translation initiation at the next AUG startcodon in an appropriate context. The availability of TC will determine how fast translation can reinitiate. Thus, eIF2 phosphorylation renders translation dependent on the distance between an uORF and the AUG start codon. Notably, the majority of transcripts that are dependent on SCF-induced PI3K activity and on availability of eIF4E in erythroblasts also contain several uORFs, including the previously mentioned transcripts encoding Nme2, Use1, and *α*4. Translation of these transcripts is hypersensitive not only to growth factor signalling but also to inhibition by oxidative stress, or iron availability.

The transcript encoding thrombopoietin (TPO) contains 7 uORFs of which the last uORF overlaps with the TPO start codon [49]. In hereditary thrombocytosis (HT) mutations that deregulate translation of some of these uORFs cause high levels of TPO expression and thrombosis.

Notably, the transcripts of several Ets-family members, Scl/Tal, and C/EBP*α* and *β* contain an upstream ORF (uORF) that overlaps and is out of frame of the proper AUG start codon of the full-length isoform of the transcription factor [50, 51]. The uORF starts with a uAUG in a suboptimal Kozak consensus and hence is only translated at enhanced activity and availability of eIF2:GTP and eIF4E. When the uORF is translated, the initiation codon of the full-length protein is skipped and reinitiation at a downstream AUG codon results in synthesis of a shorter protein isoform. In the case of C/EBP*α* translation initiation at a downstream AUG results in a truncated transcription factor protein that acts as a dominant negative isoform [52].

The importance of the relative abundance of C/EBP*α* isoforms is evidenced by the occurrence of mutations in acute myeloid leukaemia (AML) cases that inhibit translation of the full-length C/EBP*α* protein [52]. The functional 30 kDa-truncated protein expressed in these patients was shown to inhibit G-CSF receptor in 32Dcl3 cells induced to differentiate into neutrophils [53]. In addition, C/EBP*α* is required for the generation of the GMP compartment and its expression also denotes selectivity in differential commitment to monocytic lineage [54]. In a subset of AML C/EBP*α* is mutated [55]. Strikingly, two mutations are combined: an N-terminal mutation on 1 allele and a C-terminal mutation on the other allele. The N-terminal mutation abrogates the long isoform of C/EBP*α* but allows for translation from the downstream AUG start codon, which contributes to the phenotype of the AML [56].

Increasing evidence supports the importance of expressing different isoforms that modulate lineage commitment in hematopoietic cells. The expression of truncated forms of the transcription factor, Stem Cell Leukaemia (Scl), is regulated by differential initiation of translation [51] and results in erythroid lineage differentiation. Expression of functional isoforms due to differential translation has been described for other hematopoietic transcription factors [50, 57] and disruption of isoform ratios is implicated in disease [52, 53].

The list of transcripts regulated at the level of alternative AUG usage in hematopoietic regulation is increasing. A novel technique to identify translation start codons indicated that 65% of all transcripts expressed in mouse ES cells are translated from at least 2 alternative start sites [58].

 Translation initiation inhibition of growth regulatory proteins (growth factors, cytokines, oncogenes, repressors of tumour suppressor inhibitors, and others) is a known phenomenon [59] and this may be extended to regulatory proteins that attenuate cellular terminal differentiation.

### 2.3. Translation Initiation Sensitivity to eIFs: Cap-Independent Translation

**Figure 2:**
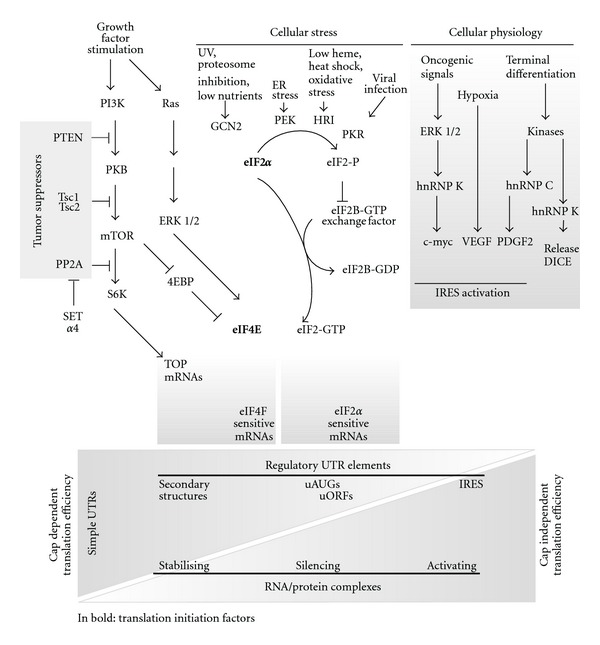
Translation initiation control during growth factor stimulation, cellular stress, and cellular physiology. Growth factor addition activates the PI3K/PKB/mTOR pathway releasing the limiting translation initiation factor 4E (eIF4E) from a repression complex with 4EBP and activating S6K resulting in enhanced cap-dependent translation efficiency of structured mRNAs and ribogenesis. Interestingly, the tumour suppressor proteins PTEN, Tsc1/2, and Pp2a are involved in attenuating this pathway. Another limiting initiation factor, eIF2*α*, is involved in providing methionine-tRNA in a complex with the 60S ribosome subunit to start peptide synthesis once the proper AUG is recognised. eIF2 is phosphorylated by GCN2, PEK, HRI, or PRK in response to various stress conditions. Low levels of eIF4E and eIF2-GTP as a result of 4EBP repression or stress-induced eIF2 phosphorylation, respectively, repress cap-dependent translation. These conditions are optimal for translation initiation from Internal Ribosomal Entry Sites (IRESs). The levels of eIFs modulate translation initiation and this depends on the codes offered by the transcripts. Some transcripts are ideal to be translated under stress conditions having IRES structures in their 5′UTRs; others have secondary structures that are difficult to melt and hence hinder the scanning process. The presence of uORFs, attenuates translation initiation and also has a role in protein isoform formation. RNA-binding proteins modulate specific mRNAs by stabilising, silencing, or activating the transcripts. These RNA/protein complexes (RNPs) have an important role in cellular physiology. Some RNPs respond to oncogenic signals, while others are covalently modified and drive translation in response to terminal differentiation signals as in the case of the DICE elements (translation initiation factors in bold).

Regulatory elements at the 5′UTR of mature transcripts render translation dependent on signalling or other environmental conditions such as iron availability. Some transcripts however have a highly structured 5′UTR meant to completely block cap-dependent translation initiation. Under the conditions that these proteins need to be synthesized, cap-dependent translation initiation is strongly decreased and translation initiation reverts to internal ribosome entry sites (IRESs). The structural complexity of IRES elements argue in favour of their role as translation inhibitors, although it is more correct to define these structures as modulators of translation. For instance, although the 5′UTR of platelet-derived growth factor (*PDGF2*) is long (1022 bp), is structured and contains upstream ORFs, it is efficiently translated during megakaryocytic differentiation via binding and activation of the IRES by hnRNP C (Figure 2) [60]. Hence, specificity of IRES-mediated gene expression is determined by IRES trans-acting factors (ITAFs), representing a particular cellular state. In addition to differentiation, translation initiation starting from an IRES is found in transcripts encoding proapoptotic proteins or proteins required specifically during G2/M when transcription is silenced by compaction of the DNA [61, 62].

IRES-dependent translation is less competitive for polysome recruitment than cap-dependent translation. Therefore, IRES-dependent translation may be preferentially impaired when ribosome subunits are reduced, a situation that is typical for Diamond Blackfan Anemia (DBA). Expression profiling of polyribosome-bound mRNAs from erythroblasts identified a specific set of transcripts that are selectively lost from polyribosomes upon reduced expression of Rps19 [63]. Among these mRNAs were transcripts encoding the Hsp70/Hsc70 cochaperone Bag1 (Bcl-2-associated athanogene 1), and the RNA binding protein Csde1 (cold shock domain containing E1), both requiring an IRES for translation initiation. Importantly, expression of BAG1 and CSDE1 was also reduced in human erythroblasts cultured from peripheral blood of DBA patients, whereas *BAG1* and *CSDE1* mRNA level was constant or even elevated. Interestingly, Csde1 binds the IRES of several transcripts and controls IRES-mediated translation [64].

### 2.4. RNA-Binding Proteins: Transcript Stability and Translation Control

mRNA translation is regulated by activities of the translation machinery components as described previously but also via regulation of proteins that bind to specific mRNAs. RNA binding protein complexes attenuate expression by modulating stability, degradation, cellular localisation, and silencing of specific RNAs or subgroups of mRNAs. The most studied RNA-binding proteins present in ribonucleoprotein (RNP) particles are the heterogeneous nuclear ribonucleoproteins (hnRNPs) that recognize AU-rich elements (ARE) and coordinate expression of mRNAs at the level of nuclear-cytoplasmic shuttling [65], cytoplasmic mRNA turnover [66], and silencing of cell state- and type-specific mRNAs [67, 68]. The ARE is located in the 3′ untranslated region of many short-lived transcripts from cytokines, proto-oncogenes, growth factors, or cell cycle regulators [69]. Interestingly, tristetraprolin (TTP) and butyrate response factor (BRF1) belong to the same protein family and both promote ARE-dependent decay [70]. Interferon *γ* (IFN*γ*) suppresses the survival and expansion of T-helper 17 (Th17) cells by inducing expression of TTP, resulting in the destabilisation of the p19 mRNA, coding for a subunit of IL23 [71, 72]. The tight regulation of proinflammatory cytokines through mRNA stability is required to suppress inflammatory responses. In fact, TTP deficient mice have an overproduction of cytokines resulting in a systemic inflammatory response with clinical outcomes including arthritis and autoimmunity [73].

In contrast to TTP and Brf1, members of the ELAV family of RNA-binding proteins (e.g., HuR) bind and stabilize ARE-containing transcripts [74]. Interestingly, in the context of a closed loop model of translated eukaryotic mRNAs, the recruitment of HuR and other RNA binding proteins to 3′UTR elements results in the formation of complexes between HuR and the scanning ribosome at the 5′UTR. This stabilises the transcript and facilitates translation initiation at the proper AUG in transcripts with a structured 5′UTR [75]. Upregulation of TNFalpha in a mouse model for autoimmune disease depends on binding of ELAV-like protein to the AU-rich elements in its 3′UTR [76].

Modification of RNA binding-proteins by signalling can be another level of regulating translation efficiency at the proper AUG. Silencing of transcripts by binding of hnRNP K and hnRNP E1 to differentiation control element (DICE) in the 3′ UTR of 15-lipoxygenase (LOX) mRNA transcript [67] can be released during terminal erythroid differentiation by phosphorylation of hnRNP K (Figure 2) [77].

Another prototype example in which RNA-binding proteins regulate translation in hematopoietic cells is the iron-response element (IRE) [78]. It is a stem-loop structure that binds iron regulatory proteins IRP1 and IRP2 dependent on their association with iron [79, 80]. Whereas the free IRE can easily be unwound by the eIF4A helicase during scanning of the preinitiation complex, IRP binding stabilises the stem-loop structure and impairs continued scanning and translation. Initially characterised for ferritin mRNA, IRE elements are found in a number of transcripts encoding proteins involved in iron metabolism or hemoglobin synthesis. An example of the latter is alpha-hemoglobin-stabilizing protein (AHSP) mRNA. Because alpha globin is synthesised ahead of beta globin, it has to be stabilised until its incorporation into haemoglobin. Regulation of AHSP by iron implies regulation of the stability of alpha globin [81].

Ceruloplasmin is involved in iron metabolism and is translationally regulated by interferon gamma [82]. Phosphorylation of ribosomal protein L13a by interferon-gamma results in dissociation of L13a from the 60S ribosome subunit and recruitment of an Rpl13-containing protein complex to a structural element in the 3′UTR of ceruloplasmin [83] resulting in translation repression. This mechanism incorporates 2 novel issues. First, the ribosome is able to present signalling sensitive factors that can be released to attenuate translation of specific transcripts without affecting global synthesis rates. Second, regulatory elements in the 3′UTR recruit protein complexes within the circular mature transcripts and interact with scanning complexes in the 5′UTR, hence modulating translation initiation efficiency.

**Figure 3:**
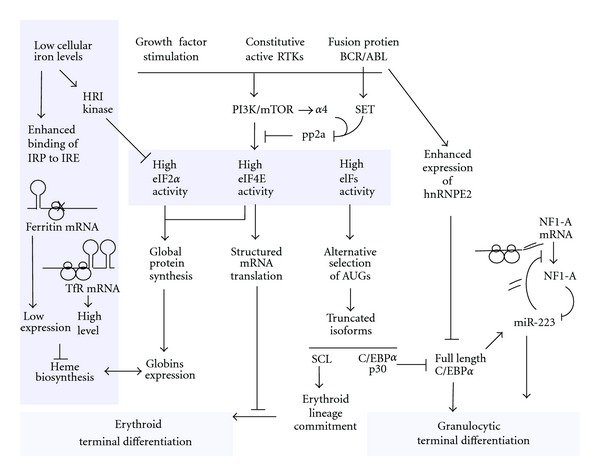
Translation initiation control relays signals to erythroid and granulocytic differentiation. SCF binds c-kit, a Receptor Tyrosine Kinases (RTKs), activating PI3K/mTOR pathway in the same way as constitutive active mutant RTKs and kinase active fusion protein, BCR/ABL. mTOR downstream effector proteins are maintained active by attenuating the phosphatase Pp2a which is inhibited by SCF-driven alpha4 expression and enhanced expression of SET in response to BCR/ABL. High activity of translation initiation factors enhances polysome recruitment of structured mRNAs and delays erythroid terminal differentiation. During erythroid terminal differentiation the balance between globin synthesis and haeme biosynthesis is under the tight control of translation initiation. Iron Responsive Element (IRE) in the UTRs of ferritin and transferrin modulates iron uptake and storage in accordance to demand of haeme. Low cellular iron levels trigger phosphorylation of eIF2*α* to reduce the production of globin proteins. High eIFs levels also regulate commitment to the erythroid or megakaryocytic lineage by selective usage of AUGs in the SCL transcript driving different isoform production. The same mechanism is used to produce truncated isoforms of the transcription factor C/EBP*α* that acts as a dominant negative form of the full length and hence inhibits granulocytic terminal differentiation. Another form of translation control is involved in regulation of C/EBP*α* transcription activity. Full-length C/EBP*α* enhances transcription of micro RNA 223 (miRNA-223), an inhibitor of NFI-A translation. NFI-A is a competitor for binding C/EBP*α* DNA sites and hence its inhibition results in a positive feedback loop driving granulocytic differentiation. In addition to transcription inhibition of full-length C/EBP*α* driven by selective AUG usage or translation silencing of competitors, the role of RNA-binding proteins is important in modulating terminal differentiation. BCR/ABL enhances the expression of hnRNPE2 that binds the UTR of C/EBP*α* transcript and inhibits translation.

The expression of RNA-binding proteins that attenuate translation of specific subsets of mRNAs has been implicated in the transition from chronic CML to blast crisis events [84, 85]. For instance, ectopic expression of hnRNP E2, an RNA-binding protein upregulated during blast crisis of CML, resulted in downregulation of C/EBP*α* (Figure 3) and G-CSFR in myeloid progenitor cells, inhibiting granulocytic differentiation [86].

## 3. Conclusion and Future Perspectives

Regulation of gene expression has been studied extensively in disease models and patient groups giving detailed annotations of the differential expression at the level of transcription regulation. Differential expression between the transcriptome and the proteome supports the importance of posttranscriptional regulation. Increasing evidence supports the prominent and so far underestimated regulation of mRNA translation, which depends on the availability and activity of the translation machinery, the structure of the transcript, and the expression of RNA binding proteins. Growth factor signaling enhances polysome recruitment of specific RNA transcripts, with marginal effect on the transcription regulation [23]. Similarly, increased availability of the translation initiation factor, eIF4E, through growth factor signaling, overexpression studies, and suppressions of the attenuation mechanism of mTOR signaling results in proliferative advantage of erythroid progenitors and block of differentiation [12, 14]. Increasingly, differential or conditional expression of transcripts are being associated with haematological malignancies, including expression of dominant negative isoforms involved in progenitor differentiation [52], expression of RNA binding proteins involved in inflammatory responses [71], and suppressed expression of phosphatase subunits resulting in progression of disease [40]. Interestingly, deregulation of negative feedback mechanisms due to low activity of phosphatases is potentially targeted using mTOR inhibitors or phosphatase activators. Hence understanding translational control mechanisms and its deregulation in disease will impact on patient stratification into therapeutic groups.
